# The Effects of Self-Esteem and Academic Engagement on University Students’ Performance

**DOI:** 10.3390/bs13040348

**Published:** 2023-04-21

**Authors:** Elizabeth Acosta-Gonzaga

**Affiliations:** Unidad Profesional Interdisciplinaria de Ingeniería y Ciencias Sociales y Administrativas, Instituto Politécnico Nacional, Mexico City 08400, Mexico; eacostag@ipn.mx

**Keywords:** self-esteem, academic engagement, academic performance, motivation, higher education

## Abstract

The success or failure of a student depends on several factors, including self-esteem, academic engagement, and motivation. Self-esteem and motivation have been found to influence academic engagement, which, in turn, contributes to academic performance. Through a quantitative study, 243 university students were surveyed to analyze the effects of self-esteem and motivation on their academic engagement, which would be reflected in their academic performance. The results show that self-esteem has effects on emotional and behavioral disengagement. Motivation shows greater effects on academic engagement, with metacognitive engagement predicting students’ academic performance. Therefore, promoting metacognitive strategies that help students learn to plan, monitor, and self-regulate their learning will contribute to their performance.

## 1. Introduction

The success or failure of a student’s learning process includes many factors; the ones that play an essential role include self-esteem, motivation, and academic engagement. System theory states that the mind is divided into three separate systems, which are the motivational, affective, and cognitive systems. The motivational system includes the basic components that make an organism aware of hunger, thirst, and even the need to reproduce in order to survive. The affective system includes emotions, such as cheerful states, enthusiasm, etc. The cognitive system comprises thought-related processes, such as reasoning, memory, and judgment, the basis for understanding the events that occur all over the world [1,2].

In the educational context, the motivational system or academic motivation is defined as an energizing catalyst that starts the action. It is also studied as a variable that is influenced by the context [3].

Academic engagement, also known as commitment (in this research, both terms are used to refer to the same concept), pushes the student to participate during educational activities [4,5]. The academic engagement level is not only crucial to ensuring that students successfully finish their learning process, but also acts as a shield against the dangers they face as young people [6]. Academic engagement has been divided into three elements for better analysis: emotional, behavioral, and cognitive [7,8,9]. Analyzing how motivation influences a student’s engagement is crucial in order to determine a student’s involvement level with the learning process, which is then reflected in terms of academic advantage; this is because it has been proven that motivation influences academic engagement, which contributes to academic achievement at the same time [10].

Self-esteem is another key factor that influences academic performance; it is relevant because it has been closely related to motivation and academic achievement [11]. Self-esteem refers to the positive or negative perception of a student’s self-worth [12,13], which affects a student’s ability to complete or not complete educational tasks. Therefore, it is essential to include this factor because it has been proven that it is positively associated with performing a task.

It is also very common for academic performance to be evaluated by looking only at the grades that the student obtains while studying, and this is seen as the golden measure of success in education [14]. It is also known that the psychosocial self-esteem factor influences grades [15,16], and that it is closely related to academic performance through emotions (affective states) and motivation [17]. Therefore, analyzing the effect of self-esteem on academic involvement will provide empirical evidence that enables the proposal of strategies that encourage appropriate levels of motivation, in order to impact educational performance positively.

Therefore, this research proposes a conceptual model that explores the effects of self-esteem, motivation, and the components of academic engagement as factors that positively influence a student’s performance. This research introduces empirical evidence for how self-esteem can affect a student’s engagement level in learning, which at the same time, is reflected in their academic performance.

## 2. Theoretical Background

### 2.1. Motivation, Academic Engagement, and Self-Esteem

In the educational field, motivation has been studied as a dependent variable that is influenced by the academic context, study field, and the task to be completed [3,18].

Several theories have been proposed in order to study the interrelation between motivation, engagement, and self-esteem, and two of them have received attention. The first one is Pintrich and de Groot’s [19] expectancy-value theory, which considers the role of motivation in fulfilling goals or tasks. These authors propose two main elements as the basis of this theory. The first one plays a crucial role in the determination of educational performance, and it refers to students’ belief in their capacity to perform educational tasks, as well the sense that they have control over and responsibility for their performance; this element was denoted as expectancy, and involves self-efficacy and students’ beliefs about their control over the learning process, which is also considered in Bandura’s [20] work. The other element was denoted as value and includes extrinsic motivation, intrinsic motivation towards the objective, and the value a student assigns to the task.

The second theory is self-determination, proposed by Ryan and Deci [21]; this states that students possess psychological needs that are provided by the motivational fundament used to prove that they are very committed to their academic activities [22]. This theory is considered the conceptual basis that explains self-esteem-related components. This theory also proposes a link between engagement, motivation, and academic achievement, and explains how changing to a new level of engagement affects motivation and the learning context.

Researchers such as Skinner and Belmont [7] and Reschly and Christenson [9] point out that academic achievement is an established goal that includes emotional, behavioral, and cognitive involvement [8].

Emotional engagement involves students’ positive and negative affective answers to the learning environment and academic tasks [8]. It includes positive emotions such as enjoyment, enthusiasm, interest, satisfaction, and vitality. The opposite behavior to involvement is called lack of interest; applied to this concept are synonyms such as disaffection or detachment. When a student is uninterested, that person shows negative emotional states, such as boredom, frustration, depression, anxiety, or even rage. Generally, unhappy students are submissive, and they do not attempt to face challenges [7].

According to González et al. [23], behavioral engagement refers to students’ participation in their academic activities, tasks, homework, and the learning environment, as well as the time and attention spent on educational tasks. In addition, Reschly and Christenson [9] and Reschly [24] mention that behavioral commitment refers to the degree to which a student participates in academic, social, or extracurricular activities, and it includes behaviors such as starting an action, making an effort to complete it, making attempts, persisting, and working with intensity, attentiveness, concentration, and participation [6]. On the other hand, behavioral disengagement includes negative behaviors that show a lack of interest in educational activities, such as passiveness, forsaking, distraction, or being mentally disconnected and unprepared [6].

Cognitive involvement implies students’ psychological investment and effort made in their learning process [23]. This is when the student moves from using simple cognitive strategies, in which their commitment is low, to using more sophisticated cognitive strategies, such as self-regulation or metacognition [25]. Metacognitive engagement is the knowledge of knowledge [26], and is found at a higher level of cognition [27,28]. When students are engaged with metacognitive actions, this leads them to self-regulated learning and to learning to plan, regulate, and monitor their knowledge. Planning helps the student to determine the best way to approach a problem. Monitoring implies being able to understand and comprehend the problem to be studied and, finally, regulating or controlling their learning [8,29].

Studying students’ involvement levels is essential determining their academic performance; this idea has been corroborated by several researchers that have suggested that student engagement is related to academic achievement [4,6,30].

Self-esteem is defined as the positive or negative attitude towards oneself. It is also considered a characteristic of liking or disliking oneself [13]. In the educational environment, it is the students’ positive or negative perception of self-value and capability [12,13]. It is also considered as the level of belief a student has in being able to obtain good grades. 

Self-evaluation comprises basic characteristics, such as self-esteem and self-efficacy [31]; both impact the wellbeing, motivation, behavior, and performance of students in the fields of educational and work [17]. In the educational environment, both constructs (self-esteem and self-efficacy) are essential characteristics that contribute to explaining individual differences in motivation, attitudes, and academic performance among students.

Self-efficacy is the trust that each individual has in achieving an objective in a particular situation; it is considered a critical element that enables students to achieve their educational goals and face decisions throughout their lifetime [32].

It has been found that self-esteem, self-efficacy, and expectations are some of the essential components that may influence student engagement, considerably affecting the quality and level of involvement [33].

Researchers [17] have found self-esteem to be closely related to affective processes, affective evaluation, or feelings, while self-efficacy has also been associated with motivational processes, motivational belief, or judgement. According to these researchers, students’ motivational and affective states are mediators that influence the relationship between self-esteem and educational performance in a different way. Therefore, self-esteem is related to academic performance through emotions (affective states) and motivation.

### 2.2. Motivation, Academic Engagement, and Self-Esteem in Academic Performance

**Figure 1:**
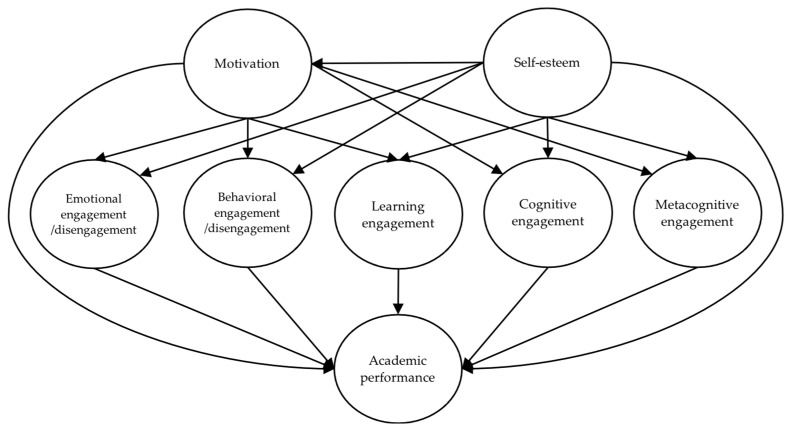
Proposed conceptual model.

The previous research mentioned below has provided empirical evidence of the relationships proposed in the conceptual model shown in Figure 1.

One of the essential components to understanding the involvement level of a student in educational activities is motivation. Many researchers have analyzed this connection and confirmed the impact of motivation on academic engagement [20,34,35]. Self-efficacy is one of the factors that comprises the construct of motivation [19]. 

Since academic engagement comprises emotional, behavioral, and cognitive elements, many researchers have analyzed these elements and motivation; for instance, Wang and Eccles [36] examined the way that motivation influences emotional, behavioral, and cognitive engagement, finding that it impacted all elements. In this way, the effects of motivation on students’ emotional, behavioral, and cognitive involvement were confirmed, as observed in the conceptual model of Figure 1.

Moreover, the influence of motivation on academic performance has been analyzed; for instance, Sun’s research [37] showed evidence of a positive connection between a motivation element (self-efficacy) and academic performance. Self-efficacy is a predictor of motivation and academic performance over time in multiple environments and populations [38].

Likewise, and by analyzing the effects of self-efficacy on academic engagement, the researcher Pellas [39] found that students who showed higher levels of self-efficacy and intrinsic value were also more likely to use cognitive and self-regulation strategies (a component of metacognitive engagement). Therefore, it is established that there is a relationship between student motivation and the metacognitive engagement involved.

It has also been found that self-efficacy is related to academic engagement, as Salamela-Aro and Upadyaya’s research [40] showed; they conducted a longitudinal study with adolescents, confirming a positive relationship between self-efficacy and academic commitment. This suggests that perceived self-efficacy influences the degree of commitment and effort a student invests when performing a task [20,41].

### 2.3. Effects of Self-Esteem in Emotional, Behavioral, Cognitive, and Metacognitive Engagement

According to Griffiths and colleagues [33], some essential elements that may influence student engagement are self-esteem, self-efficacy, expectations, and relations among peers [20], all of which substantially affect the quality and level of involvement.

Previous researchers have discovered that self-esteem can predict academic performance [15,16]. Therefore, there is not only a direct relationship, but also a reciprocal connection, meaning that the school grade positively predicts self-esteem [42].

Pellas’ research [39] analyzed how computer self-efficacy, metacognitive self-regulation, and self-esteem influenced students’ academic engagement [43] in online careers at university. His discoveries revealed that self-esteem, computer self-efficacy, and self-regulation were meaningful predictors of students’ cognitive engagement but not of their behavioral engagement, which was negative. The research also showed that students’ self-esteem was not only positively correlated with emotional and cognitive engagement, but also negatively correlated with behavioral engagement elements.

Regarding emotional and behavioral engagement, van der Kaap-Deeder et al. [44] carried out two studies to determine the relationship between self-esteem, motivation, and engagement. Their results showed a positive relationship between self-esteem and emotional and behavioral engagement, as well as emotional disaffection and anxiety towards exams. Moreover, Zeigler-Hill et al.’s [45] study showed that unstable self-esteem was related to academic disaffection. 

The relation between self-esteem and metacognitive engagement has also been verified, as shown in a study by Du and colleagues [46], who analyzed how students used self-regulated learning (considered a metacognitive strategy) to solve a specific task. Their results confirmed that self-esteem was a factor influencing students’ ability to self-regulate learning. 

A study by Zuffianò et al. [47] found that self-esteem was not related to academic performance, suggesting that this was because the construct used in their research assessed self-esteem in general, and that, in fact, a more specific construct called academic self-esteem should be proposed.

Regarding behavioral engagement, previous educational research has shown that it significantly affects academic performance [8,48,49].

In the same way, studies have shown that emotional engagement has an effect on academic performance [50,51]. For instance, Tze et al. [52] conducted a meta-analysis that analyzed the relationship between academic boredom, motivation, and academic performance. Their findings showed that boredom in class had more adverse effects on academic performance than boredom while studying.

Some of the literature has analyzed the influence of cognitive engagement on academic performance [19,53,54]. For instance, Broadbent’s study [54] found that students who applied cognitive strategies, such as elaboration and time management, had better educational grades.

On the other hand, the research by Chen and Wu [55] confirmed that there was a relationship between metacognitive engagement and students’ academic performance.

The works described above provide evidence for the relationships proposed in the conceptual model shown in Figure 1.

## 3. Materials and Methods

### 3.1. Data Collection

The sample was obtained from 243 university students who were told about the study’s objectives and then decided to participate voluntarily; they were told that they could leave the study at any time, and the students agreed to their answers for each item being be used in this study [56]. The sample was collected from students undertaking degrees in sciences and social sciences enrolled in either industrial management, computer science or industrial engineering courses at a public university. The sample included students from all semesters. They were surveyed online during the second semester of the 2020 school year. The participants’ ages were between 19 and 21; 108 were male and 134 were female. Their teachers reported the students’ final grades after the last examination, which were used to measure academic performance (AP). The final grade ranged from 1 to 10.

To calculate the causal relations in the theoretical model, Structural Equation Modeling (SEM) was used. SEM analysis was conducted using the partial least squares method, using the software SmartPLS version 3 (SmartPLS GmbH, Oststeinbek, Germany).

The general rule of sample size was considered for calculating the model quality. Wang and Wang [57] suggest that the sample size depends on the number of indicator variables (items). They mention that five cases per indicator variable are sufficient. The number of students that answered all the items was 243, and 30 was the number of indicator variables in the analysis SEM. Therefore, the sample size was sufficient to reach the quality of the model in this study.

### 3.2. Instruments

The Rosenberg [12] self-esteem scale was used in this research, which measures how a person evaluates their worth as a human being. The scale has been widely tested in different studies showing good psychometric characteristics [58], and has also been validated in the Mexican context [59]. This scale contains the same number of positive and negative questions. An example of a positive question would be “overall, I am satisfied with myself”; a negative one would be “I feel like I don’t have much to be proud of”. Negative questions were recorded, so that a lower number indicated a higher level on the scale. Items were evaluated using the Likert scale from 1 to 5, used the following rankings: 5 = totally agree, 4 = agree, 3 = neither agree nor disagree, 2 = disagree, 1 = totally disagree.

The Motivated Strategies for Learning Questionnaire (MSLQ) by Pintrich [60] and Pintrich and de Groot [19] was used to evaluate the motivational, cognitive, and metacognitive factors of the students, which were estimated using the expectation and value components. The first component included self-efficacy (SE), control learning beliefs (CLB), and test anxiety (TA). The value component included intrinsic goal motivation (IGM), extrinsic goal motivation (EGM), and beliefs about the importance of a task or the task value (TV).

The MSLQ also captured learning strategies that encompassed help-seeking (HS) and peer learning (PL). Cognitive strategies included organization (OR), elaboration (EL), and rehearsal (RE). Metacognitive strategies included self-regulation (SR) and critical thinking (CT). The response to the instrument was answered using a Likert scale between 1 (not at all true of me) and 7 (very true of me). This instrument has been used and validated in the Mexican context [27,28,61].

In the same way, Skinner et al.’s [6] instrument, Student Engagement and Disaffection in school (SED), was used. The scale measures emotional engagement and disengagement, as well as behavioral engagement and disengagement. Emotional engagement included enjoyment (EN), enthusiasm (ET), fun (FU), pride (PR), and interest (IN). Emotional disengagement included boredom (BO), disinterest (DI), frustration (FR), sadness (SA), and anxiety (AN). Behavioral engagement included involvement (IN), effort (EF), and attention (AT). Behavioral disengagement included being distracted (DI), mentally withdrawn (ME), and passive (PA). As with the MSLQ, this instrument has been previously used in studies by [27,28,61]. The scale was evaluated on a 5-point Likert scale, where 1 meant completely disagree, and 5 meant completely agree.

The SED and MSLQ instruments were applied mid-semester, and the self-esteem instrument was applied at the end of the semester.

### 3.3. Data Analyses

The reliability of the items was verified before using the SEM method. The reliability analysis was measured using Cronbach alpha values; despite there being no minimum value universally accepted, many authors suggest that this must be higher than 0.70 [62,63]. 

The convergent validity was verified through the composite reliability (CR) and average variance extracted (AVE). Composite reliability values up to 0.7 and 0.5, respectively, are considered acceptable [64]. Discriminant validity was verified through the Fornell–Larcker [65] criterion; it was verified when the square root of the AVE values was higher than the correlation among the rest of the constructs.

## 4. Results

### 4.1. Reliability and Validity

After analyzing the theoretical model using the Structural Equation Modeling technique, the validity of the constructs was verified. Therefore, the enthusiasm (ET) and pride (PR) variables were removed from emotional engagement. Test anxiety (TA) was removed from motivation. Table 1 shows the acceptable values using the Cronbach’s alpha, CR, and AVE of the resulting constructs, which shows that the construct validity of the structural model was reached.

Table 2 shows the discriminant validity values, in which the values below the diagonal are lower than the ones crosswise (in bold); therefore, the discriminant validity of the theoretical model was reached.

Table 2 shows that the correlation between the constructs was lower than the square root of the AVE values; therefore, discriminant validity was reached.

### 4.2. Causal Model

**Figure 2:**
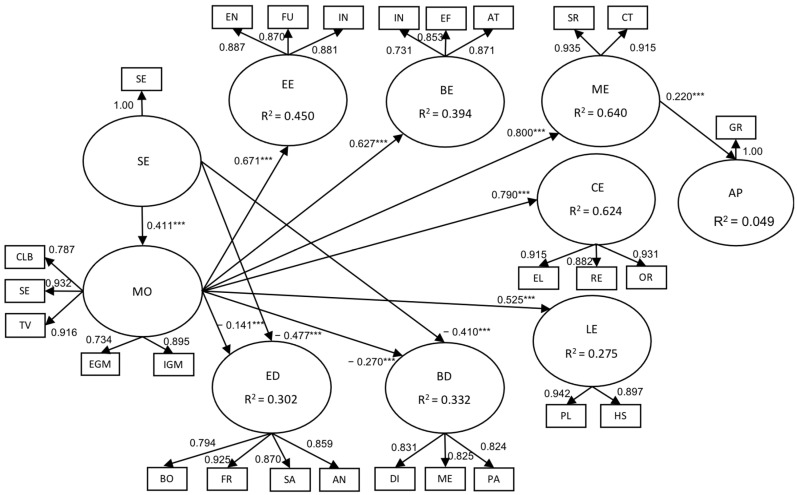
Significant relationships of the causal model *** *p* < 0.001.

Figure 2 shows the resulting model, in which the causal relationships that were confirmed from the proposed conceptual model are observed.

The results revealed a negative relationship between self-esteem and emotional and behavioral disengagement. At the same time, it was notable that motivation played a dominant role in the resulting model by showing effects on emotional, behavioral, cognitive, metacognitive, and learning engagement. Among the elements that comprise motivation, self-efficacy and the task value were the ones that showed higher effects.

Moreover, we discovered the existing positive connection between metacognitive engagement and academic performance; in contrast, self-esteem had no effects on emotional, behavioral, learning, cognitive, and metacognitive engagement, or on academic performance. 

## 5. Discussion

For students, self-esteem is the subjective evaluation of their worth, or the positive or negative attitude they have towards themselves [12]. This perception can contribute to how much students believe in their own academic ability. The result of this research showed that self-esteem had effects on emotional and behavioral disengagement. This result may be due to the fact that the level of self-esteem observed in the sample was M = 2.49, evaluated on the Likert scale from 1 to 5.

Those students who do not believe in their abilities may experience emotional disengagement, including boredom, frustration, sadness, and anxiety. Moreover, they may manifest behavioral disinterest with attitudes such as distraction, mental detachment, and passivity. This result agreed with the research by Zeigler-Hill et al. [45], who showed that unstable self-esteem was related to high levels of academic disinterest. 

This study revealed no relationship between self-esteem and academic performance, which coincided with other investigations that have found that this may be because other factors affect this relationship [66] or that the self-esteem construct is a weak predictor [16]. 

Students who have a positive evaluation of themselves, that is, high self-esteem, can pour this positive evaluation into their school activities and believe that they can also perform well [66]. On the contrary, this research found that the students had low levels of self-esteem. This could mean that it had no direct correlation with academic performance, which would coincide with Hyseni Duraku and Hoxha [67], who proposed that the evidence regarding the effect of self-esteem on academic performance in higher education students is controversial.

This study also found that motivation affected the emotional, behavioral, cognitive, and metacognitive engagement of students, as well as their emotional and behavioral disengagement. This finding coincided with Martin et al. [34] and Sinatra et al. [35], who mentioned that motivation was essential in order to explain academic engagement.

The results showed that there was a strong correlation between students’ motivation and emotional commitment, which means that motivated students showed interest and enthusiasm in their classes and even had fun. This relationship was also found in the investigations of Skinner et al. [30] and Wang and Eccles [36]. Further, the study by Acosta-Gonzaga and Walet [68] also showed that fun was an essential factor in learning mathematics through online exams.

In a similar way, it was found that there was a strong relationship between motivation and behavioral engagement, so that committed students paid attention in their class, made an effort, and became involved in their activities [30,36,69].

However, it was also found that motivation influenced emotional [52] and behavioral disengagement. Although its effects were lesser than emotional and behavioral engagement, this result shows that students may feel boredom, frustration, and even anxiety in their classes. They also may display negative behaviors such as distraction or passivity.

Motivation’s effects on cognitive engagement have been proven. Motivated students apply more complex cognitive strategies, such as organization and elaboration, instead of simple strategies [70], proving the link between motivation and cognitive engagement [36].

Moreover, this research demonstrated a relationship between motivation and learning strategies. This coincided with Zhang et al.’s [71] research, which found that learning strategies were mediators between motivation and vocabulary acquirement (educational performance).

The effects of motivation towards metacognitive engagement were also verified; this same relationship was found by Butz et al. [72], who found that motivation influenced cognitive processes, mentioning that students who reported higher levels of self-efficacy and intrinsic value (motivation factors) also reported high levels of the use of cognitive and metacognitive strategies (self-regulation).

In the same way, the relation between metacognitive engagement and academic performance was verified [55], suggesting that students used metacognitive strategies such as critical thinking and self-regulation, which influenced their academic performance. Therefore, motivated students tend to plan, monitor, and self-regulate their learning process. Planning helps them to determine how to approach the problem. Monitoring implies understanding the topic to be studied, thus regulating their learning [8,29].

The results of this study were consistent with the research by Fredricks et al. [8], who found that students’ academic performance was enhanced when motivation was involved in emotional, cognitive, and behavioral engagement.

## 6. Conclusions

Whether or not students achieve their academic goals depends on several personal factors. Therefore, this study investigated the effects of general self-esteem and academic motivation on academic performance. Although controversial results were found in the self-esteem study, it was thought this would reveal effects on cognitive strategies, academic commitment, and academic performance. The results showed effects on the emotional and behavioral disinterest of the student; that is, when students do not fully trust their abilities to perform their academic tasks, they show emotions such as boredom, anxiety, sadness, or frustration, and exhibit behaviors such as passivity, distraction, and mental detachment. This implies that school administrators and teachers could propose actions that help promote the personal worth of the student. For instance, they could recommend successful academic experiences to students, and teachthem how to manage anxiety and stress.

However, it was observed that academic motivation affects the components of school engagement, so it can be assumed that motivated students will be more engaged and have better academic achievement. Motivated students show skills such as self-efficacy and intrinsic worth, and report metacognitive skills such as critical thinking and self-regulation, which influence their academic performance. Teachers could provide master classes to students by giving exceptional examples of success during their learning in order to promote students’ motivation.

### 6.1. Limitations of the Research

Some limitations are considered. For instance, academic engagement was measured using students’ self-reports, and the sample size was gathered from a single university.

### 6.2. Future Research

Future work could consider analyzing the academic self-esteem and self-concept constructs in a conceptual model for a comparative study of public and private university students, including a larger sample size.

## Figures and Tables

**Table 1:** CR, AVE, and Cronbach’s alpha of the constructs of the causal model.

Construct	CR	AVE	Cronbach’s Alpha
Self-esteem (SE)	1	1	1
Motivation (MO)	0.932	0.733	0.907
Emotional Engagement (EE)	0.911	0.774	0.854
Emotional Disengagement (ED)	0.921	0.745	0.886
Behavioral Engagement (BE)	0.860	0.673	0.759
Behavioral Disengagement (BD)	0.866	0.684	0.769
Metacognitive Engagement (ME)	0.922	0.856	0.832
Cognitive Engagement (CE)	0.935	0.827	0.896
Learning Engagement (LE)	0.916	0.846	0.821
Academic Performance (AP)	1	1	1

**Table 2:** Discriminant validity of the constructs of the causal model.

Construct	SE	MO	EE	ED	BE	BD	ME	CE	LE	AP
SE	**1.00**									
MO	0.411	**0.856**								
EE	0.335	0.335	**0.88**							
ED	0.535	0.336	0.382	**0.863**						
BE	0.424	0.627	0.694	0.339	**0.821**					
BD	0.521	0.438	0.481	0.744	0.525	**0.827**				
ME	0.269	0.8	0.578	0.293	0.638	0.456	**0.925**			
CE	0.324	0.79	0.596	0.257	0.576	0.435	0.836	**0.909**		
LE	0.269	0.525	0.355	0.096	0.496	0.264	0.603	0.53	**0.920**	
AP	0.091	0.221	0.128	0.114	0.180	0.167	0.219	0.216	0.082	**1.00**

Self-esteem (SE), Motivation (MO), Emotional Engagement (EE), Emotional Disengagement (ED), Behavioral Engagement (BE), Behavioral Disengagement (BD), Metacognitive Engagement (ME), Cognitive Engagement (CE), Learning Engagement (LE) and Academic Performance (AP). The values in bold are the square-root of AVE of each of the constructs.

## Data Availability

Not applicable.
